# Characterization of a novel GH10 alkali-thermostable xylanase from a termite microbiome

**DOI:** 10.1186/s40643-022-00572-w

**Published:** 2022-08-17

**Authors:** Maria Laura Mon, Rubén Marrero Díaz de Villegas, Eleonora Campos, Marcelo A. Soria, Paola M. Talia

**Affiliations:** 1 Instituto de Agrobiotecnología y Biología Molecular (IABIMO), Instituto Nacional de Tecnología Agropecuaria (INTA), Consejo Nacional de Investigaciones Científicas y Técnicas (CONICET), Hurlingham, Buenos Aires, Argentina; 2 Facultad de Agronomía, Cátedra de Microbiología Agrícola, Universidad de Buenos Aires, INBA UBA-CONICET, Ciudad Autónoma de Buenos Aires, Argentina

**Keywords:** GH10, Endoxylanase, Biochemical characterization, Termite gut microbiome

## Abstract

The aim of the present study was to assess the biochemical and molecular structural characteristics of a novel alkali-thermostable GH10 xylanase (Xyl10B) identified in a termite gut microbiome by a shotgun metagenomic approach. This endoxylanase candidate was amplified, cloned, heterologously expressed in *Escherichia coli* and purified. The recombinant enzyme was active at a broad range of temperatures (37–60 ºC) and pH values (4–10), with optimal activity at 50 ºC and pH 9. Moreover, its activity remained at more than 80% of its maximum at 50 °C for 8 h. In addition, Xyl10B was found to be stable in the presence of salt and several ions and chemical reagents frequently used in the industry. These characteristics make this enzyme an interesting candidate for pulp and paper bleaching industries, since this process requires enzymes without cellulase activity and resistant to high temperatures and alkaline pH (thermo-alkaliphilic enzymes). The products of xylan hydrolysis by Xyl10B (short xylooligosaccharides, xylose and xylobiose) could be suitable for application as prebiotics and in the production of bioethanol.

**Graphical Abstract:**

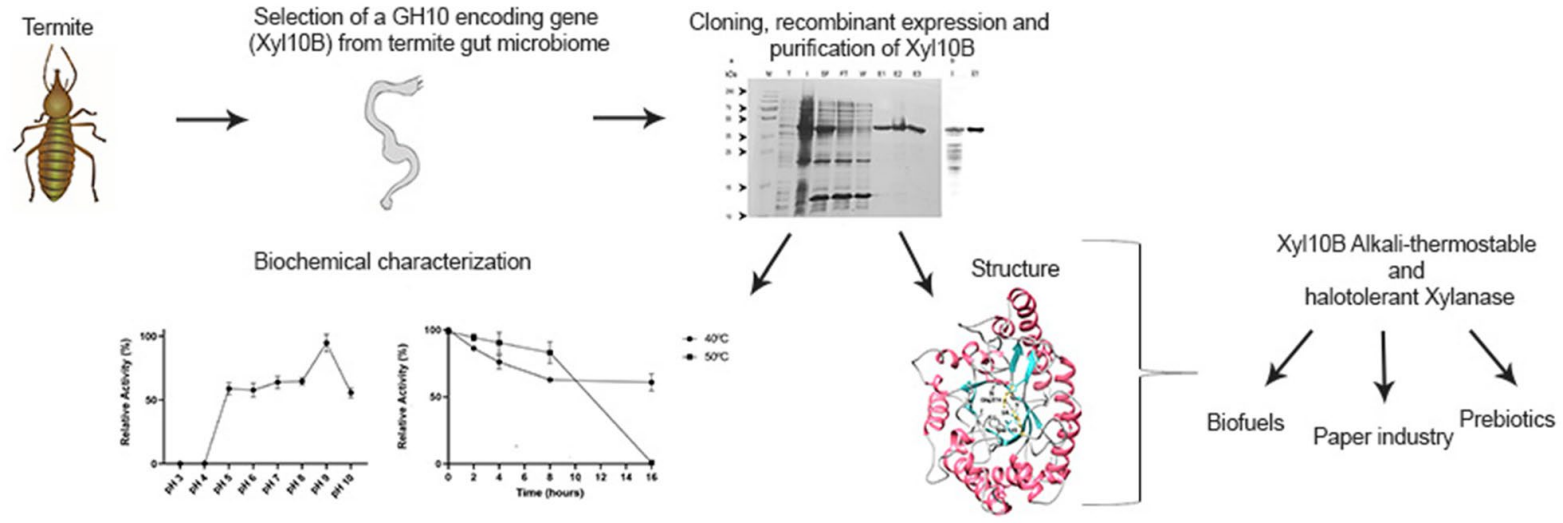

**Supplementary Information:**

The online version contains supplementary material available at 10.1186/s40643-022-00572-w.

## Key points

1. Biochemical and molecular structural characterization of a novel GH10 xylanase (Xyl10B) from a termite gut microbiome.
2. Xyl10B is a candidate biocatalyst in the bleaching process of pulp and the paper industries because of its inactivity on carboxymethyl cellulose.
3. The shorter xylooligosaccharides generated from the hydrolysis of xylan would be suitable in different applications, including the food industry as prebiotics.

**Supplementary Information:**

The online version contains supplementary material available at 10.1186/s40643-022-00572-w.

## Introduction

A sustainable promising alternative for the production of bio-products, such as biofuels, food additives, prebiotics and enzymes is the use of the lignocellulosic biomass (Adegboye et al. 2021; Gabanelli et al. 2021; Vasić et al. 2021).

Lignocellulosic biomass is mainly composed of three major polymers: cellulose, hemicellulose and lignin. Hemicellulose represents the second most abundant renewable polymer present in plant cell walls after cellulose (Narisetty et al. 2021). Among hemicellulases, xylanases (EC 3.2.1.8) are responsible for the degradation of xylan backbones into short xylooligosaccharides by a general acid–base mechanism that involves two glutamic acid residues. These enzymes are classified into glycoside hydrolase (GH) families and belong to some of the following families: GH5, GH8, GH10, GH11, GH30 and GH141, according to amino-acid sequence similarities (Verma 2021).

In the last years, some novel xylanases with desirable properties for biotechnological applications have identified and characterized by following two metagenomic approaches: functional metagenomic libraries (Mo et al. 2010; Alvarez et al. 2013; Ellilä et al. 2019; Liu et al. 2019; Alves et al. 2020; Wu et al. 2021b), and shotgun metagenomic sequencing (Joshi et al. 2020; Romero Victorica et al. 2020; Dao et al. 2021; Liew et al. 2022). Several xylanases are present in a variety of microorganisms from different environments; however, most of these enzymes lack adequate characteristics to withstand the extreme conditions of the industrial processing of lignocellulosic materials (Verma 2021). Besides, different industrial processes require specific characteristics for the same type of enzyme. For example, the biobleaching processes of the pulp and paper industries require thermo-alkali-stable xylanases (Bhardwaj et al. 2019; Mhiri et al. 2020), while the bread, juice, and feed industries prefer xylanases and β-xylosidases with thermo-acid stable properties (Verma 2021). The seafood industry, on the other hand, require salt-tolerant hemicellulases (Al-Darkazali et al. 2017).

Most of the hemicellulases available in the market to date are acidic and come from fungi. However, several of these fungal enzymes have high molecular weight and short life span at acidic environments and, for these reasons, researchers have searched for acidiphilic and alkaliphilic xylanases in other organisms, such as bacteria and archaea (Verma and Satyanarayana 2020). Bacterial xylanases have low molecular weight, present enzymatic activity under broad temperature and pH ranges, and display longer stability than fungal xylanases. These characteristics of bacterial xylanases allow rapid diffusion into the rigid lignocellulosic biomass. In addition, some of these xylanases are not active on cellulose, which is a highly desirable characteristic for some industries, including the pulp and paper industries. Finally, the easy culture and rapid harvesting of bacterial cells make bacteria an attractive choice for enzyme production (Verma and Satyanarayana 2020).

The development of novel enzymes is highly dependent on the screening strategy and the diversity of the microorganisms present at a given environment (Batista-García et al. 2016; Adegboye et al. 2021). In the case of termites, their efficiency in lignocellulosic degradation relies on both endogenous and exogenous enzymes produced by a diversity of microorganisms hosted in their intestines (Dheeran et al. 2012; Ben Guerrero et al. 2015; Batista-García et al. 2016; Vikram et al. 2021).

In a previous study, through a shotgun approach to analyze microbiomes obtained from the gut of two termite species, we identified some bacterial candidate genes coding for enzymes with lignocellulose-degrading features (Romero Victorica et al. 2020). With those results in mind, the aim of the present study was to in silico scan the metagenomic contigs to assess possible alkali-thermostable enzymes, and thereafter clone and express as a recombinant, a GH10 endoxylanase candidate (Xyl10B) to perform biochemical and molecular structural characterizations.

## Materials and methods

### Identification and selection of a GH10-enconding gene from a termite gut microbiome

Our previous studies on contigs constructed using shotgun metagenomic sequencing from termite gut microbiomes revealed that GH10 is the most abundant family involved in hemicellulose degradation (Romero Victorica et al. 2020). From the contigs assembled, we selected a predicted GH10-encoding gene KBCPBGKF_14734 (hereafter termed as Xyl10B), according to the criteria described in that study.

### Sequence analysis and molecular modeling of Xyl10B

An initial sequence analysis of Xyl10B was performed with Basic Local Alignment Search Tool for protein sequences (BLASTP) against the RefSeq protein collection of the NCBI database. The Xyl10B amino acid sequence was then aligned with 32 bacterial GH10 reference sequences with BLASTP. The expected value used was < 1E^−64^, available in the NCBI database, and the Muscle method was used as implemented in the MEGA X software (v. 10.2.6) (Kumar et al. 2018). Multiple alignment was used to calculate a Jones-Taylor-Thornton (JTT) substitution model for proteins and therefore build a maximum likelihood phylogenetic tree with 1000 bootstrap replicates using MEGA X.

Molecular models of the Xyl10B structure were developed with the I-TASSER server (Roy et al. 2010) (http://zhanglab.ccmb.med.umich.edu/I-TASSER/) and the AlphaFold software, using its online Colab notebook (Jumper et al. 2021). AlphaFold improves the accuracy of structure predictions by incorporating novel neural network architectures and training procedures based on the evolutionary, physical and geometric constraints of protein structures. For the model refining stage, we used FoldX software routines (script RepairPDB; http://foldx.crg.es/). FoldX identifies the residues that have bad torsion angles or Van der Waals' clashes and we repair them, and explores different rotamer combinations to achieve new minimum energy (Schymkowitz et al. 2005). The quality of the models was evaluated at the SWISS-MODEL Workspace (Structure Assessment tool) using QMEAN (Benkert et al. 2011) and MolProbity (Chen et al. 2010) (https://swissmodel.expasy.org/qmean/). Model manipulation and imaging were developed using the Chimera software (Pettersen et al. 2004).

### Cloning, heterologous expression and protein purification of Xyl10B

Total DNA was extracted from *N. aquilinus* gut samples by using the QIAamp DNA Stool kit (Qiagen) following the manufacturer's indications with modifications. Briefly, pooled gut samples from six individuals were heated at 95 °C in 1 mL of lysis buffer, and then disrupted with a FastPrep protocol (3 cycles of 20 s at 6,000 rpm) using glass beads (300 mg; 150–212 μm beads; Sigma, USA). An additional magnetic purification step was performed with 1.5 volumes of magnetic bead solution (Agencourt AMPure XP magnetic beads, Beckman Coulter, USA) for 5 min, followed by two 80% ethanol washes.

The Xyl10B sequence was amplified, without the native signal peptide, with specific primers (designed from the contig sequence assembled) containing the *Bam*HI and *Xho*I restriction enzyme sites (Xyl10B-F: 5′ GGATCCTACAACGCCCCCG 3′, Xyl10B-R: 5′ CTCGAGTTACTTAACCAGTTCCC- 3′), to subsequently perform an N-terminal fusion to a 6xHis tag (restriction sites are shown underlined). The amplification product was first cloned into a pGEM-T Easy vector, using *E. coli* DH5-α competent cells. Then, the plasmid inserts from positive colonies were subcloned into a pET28b ( +) vector (*Bam*HI/*Xho*I) (Novagen, United Kingdom) and transformed into competent *E. coli* Rosetta cells (DE3) (Novagen, United Kingdom).

Xyl10B protein expression was induced with 1 mM isopropyl-β-D-thiogalactopyranoside (IPTG) for 16 h at 28 °C. The cells were subjected to lysis and sonication (six pulses of 10 s, 28% amplitude) and the recombinant protein was purified in the soluble fraction with a Ni-NTA agarose resin (Qiagen), using 50 mM NaH_2_PO_4_, 300 mM NaCl, and 250 mM imidazole, at pH 8, as elution buffer. The concentration of the purified protein was estimated using Bradford Reagent (Biorad) with bovine serum albumin (BSA) as standard. This process allowed producing 2.6 mg of purified soluble active recombinant protein from 50 mL induced *E. coli* cultures.

### Protein electrophoresis and Western blot assays

The molecular weight of protein fractions was assessed with standard sodium dodecyl sulphate polyacrylamide gel electrophoresis (SDS-PAGE). The purified enzyme fractions mixed with the same volume of cracking buffer 2X were boiled at 100 °C for 5 min and electrophoretically separated on 12% SDS–PAGE. The proteins fixed in the gels were visualized with Coomassie Brilliant Blue R-250 and de-stained with discoloring solution (50% methanol, 10% acetic acid and 40% water) for 3 h.

Western blotting was performed by blotting SDS–PAGE- separated proteins onto a Hybond C-Extra nitrocellulose membrane (GE Healthcare Life Science, USA) by using Mini Trans-Blot™ (BioRad, Irvine, USA) according to the manufacturer’s specifications. The recombinant proteins were detected with the anti-His mouse antibody (GE Healthcare Life Science, USA) and the anti-mouse alkaline phosphatase conjugated goat antibody (Sigma-Aldrich, USA), using the BCIP/NBT substrate. A prestained Page ruler Plus protein ladder (10–250 kDa) (Thermo Scientific, USA) was used as a molecular weight marker.

### Enzymatic assays

The endoxylanase and endoglucanase activities of Xyl10B were assessed in 1% (w/v) of beechwood xylan and 1% (w/v) carboxymethyl cellulose (CMC), respectively (Sigma-Aldrich, USA) in a final reaction volume of 0.1 mL, at 50 °C and 400 rpm for 20 min in a Thermomixer (Eppendorf, Germany). The reducing sugars released from the hydrolysis of xylan and CMC were measured using the 3,5-dinitrosalicylic acid (DNS) assay at 540 nm with xylose or glucose as standards, respectively. The reaction was adapted to small volumes (Romano et al. 2013).

The arabinofuranosidase, β-glucosidase and xylosidase activities of Xyl10B were evaluated using, 4-nitrophenyl α-L-arabinofuranoside (*p*NPA) (Megazyme, Ireland), 4-nitrophenyl-β-D-glucopyranoside (*p*NPG) and 4-nitrophenyl-β-D-xylopyranoside (*p*NPX) (Sigma-Aldrich, USA). Reactions of 0.1 mL containing 2.5 mM of each substrate were prepared in 50 mM sodium phosphate buffer (pH 8) and the properly diluted enzyme solution. The mixtures were incubated at 50 °C for 20 min and, subsequently, the reaction was stopped by adding 0.5 mL of 2% Na_2_CO_3_. The concentration of the released *p*-nitrophenol (*p*NP) was calculated according to a standard curve by measuring the absorbance at 410 nm. The enzyme activities were expressed as IU/mg of protein. One international unit (IU) was defined as the amount of enzyme that released 1 μmol of product per minute under the conditions specified for all enzymatic assays.

### Optimum pH, temperature and thermal stability

The optimal pH was assessed using sodium citrate (pH 3–4), sodium phosphate (pH 5–8) and glycine–NaOH (pH 8.5–10) buffers at 50 °C. The influence of temperature was evaluated by incubation at pH 9 and at temperatures ranging between 20 °C and 70 °C.

The thermal stability was assessed by pre-incubating the enzymes at 40 °C and 50 °C from 0 to 16 h. In addition, kinetic parameters were determined under optimal assay conditions using 0–50 mg/mL of beechwood xylan as substrate, by fitting models to data with the GraphPad Prism software v 8.0 (http://www.graphpad.com/scientific-software/prism/).

### Effects of metal ions and reagents

The effects of various metal ions (CaCl_2_, CuSO_4_, NiCl_2_, MgCl_2_, MnSO_4_, and ZnSO_4_ at 1 mM and 10 mM), chemical reagents (ethylenediaminetetraacetic acid (EDTA), SDS, Tween-40, dimethyl sulfoxide (DMSO), and β-mercaptoethanol at 1 mM, 10 mM or 0.5%) and NaCl (1 M, 3 M and 5 M) on Xyl10B activity were determined by adding the individual reagent in sodium phosphate buffer (pH 9.0) into the standard reaction and incubating at 50 °C for 20 min. Beechwood xylan (1%) was used as substrate. The activity observed in the absence of metal ions or reagents was considered as 100% (control).

### Determination of the mode of action of Xyl10B and ligand interaction analysis

The hydrolysis patterns were qualitatively analyzed by thin layer chromatography (TLC) according to Ben Guerrero et al. (2020). After performing the enzyme reactions over different times in 100 µL reaction mixtures with 1% beechwood xylan (20 min, 40 min, 1 h, 2 h, 3 h and 16 h), we carried out the TLC technique, where the plate with the samples was placed in a chamber with ethanol/acetic acid/water as solvents in a proportion of 2:2:1 as mobile phase for 2 h. Later, the plates were revealed using water/ethanol/sulfuric acid in a proportion of 20:70:3, with orcinol solution 1% (v/v) as development system with spray and heat-gun. The hydrolysis patterns were compared with the following xylooligosaccharides as standards: xylose (X1) (Sigma-Aldrich, USA), xylobiose (X2), xylotriose (X3), xylotetraose (X4) and xylopentaose (X5) (Megazyme, Ireland). The mode of action of Xyl10B was evaluated by using X2, X3, X4 and X5 (10 mM), and beechwood xylan (1%) and then subjecting the hydrolysates to TLC. Both assays were performed at 50 ºC and pH 9.

The in silico ligand interaction analysis was implemented starting from a structure alignment of Xyl10B with a specific Protein Data Bank (PDB) model for the GH10 CbXyn10C complexed with xyloheptaose (PDB 5OFK) and using the Chimera software (Pettersen et al. 2004). This task positioned the exogenous ligand at the Xyl10B enzymatic groove and, after the removal of the CbXyn10C, the remaining Xyl10B-xyloheptaose complex was energetically optimized in its interacting atoms. The resulting complex was uploaded at the PDBsum server (Laskowski et al. 2018), which runs a program for automatically plotting protein–ligand interactions (LIGPLOT) (Wallace et al. 1995).

### Statistical analysis

All the assays were carried out in triplicate. Two independent biological replicate assays were performed with equivalent results. The results are expressed as the mean ± one standard deviation of the triplicate measurements.

To assess the reproducibility of the enzyme activities we considered the measurements for the control treatments of four independent experiments. In the case of thermostability experiments, we also included data with up to 2 h of exposure at 40 °C and 50 °C. The standard conditions for the control treatments were pH 9 and 50 °C. The coefficient of variation was 17.5% and non-significant statistical differences among experiments were observed for the control treatments (ANOVA, *P* = 0.032) (Additional file 1: Fig. S1).

### Nucleotide and amino acid sequence accession numbers

The nucleotide and amino acid sequences of Xyl10B were deposited in the GenBank database, with accession numbers OK617332 and UKT5974, respectively.

## Results

### Amino acid sequence, phylogenetic analysis and structural predictions for Xyl10B

According to the BLASTP analysis, the amino acid sequences most similar to Xyl10B were that of a GH10 endo-1,4-β-xylanase from *Treponema azotonutricium* (WP_015712816.1) (45% identity, E value 2e^−96^, and 99% coverage) and a GH10 endo-1,4-β-xylanase from an uncultured bacterium contig obtained from the gut microbiome of a wood-feeding higher termite (AGS52346.1) (49% identity, E value 6e^−107^, and 95% coverage).

**Fig. 1:**
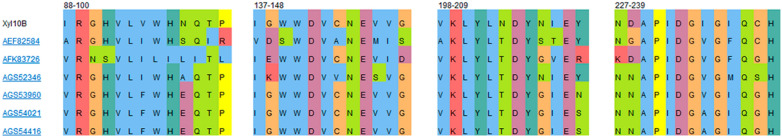
Interpro web analysis of the amino acid sequence of Xyl10B. The figure depicts the positions of four matches of Xyl10B to the glycosyl hydrolase family 10 signature (PR00134) in the PRINTS-S database and the alignment of the six most similar sequences retrieved from the GH10 accessions deposited in the CAZy database. The coordinates of Xyl10B corresponding to these matches are shown above each block

According to a functional query based on the amino acid sequence of Xyl10B in the Interpro database, the sequence highly matched the GH10 consensus at the levels of family, domain and motif in several databases. Figure 1 depicts the positions of four matches of Xyl10B to the glycoside hydrolase family 10 signature (PR00134) in the PRINTS-S database and the alignment of the six most similar sequences retrieved from the GH10 accessions deposited in the CAZy database. The coordinates of Xyl10B that correspond to these matches are indicated above each block (Fig. 1).

**Fig. 2:**
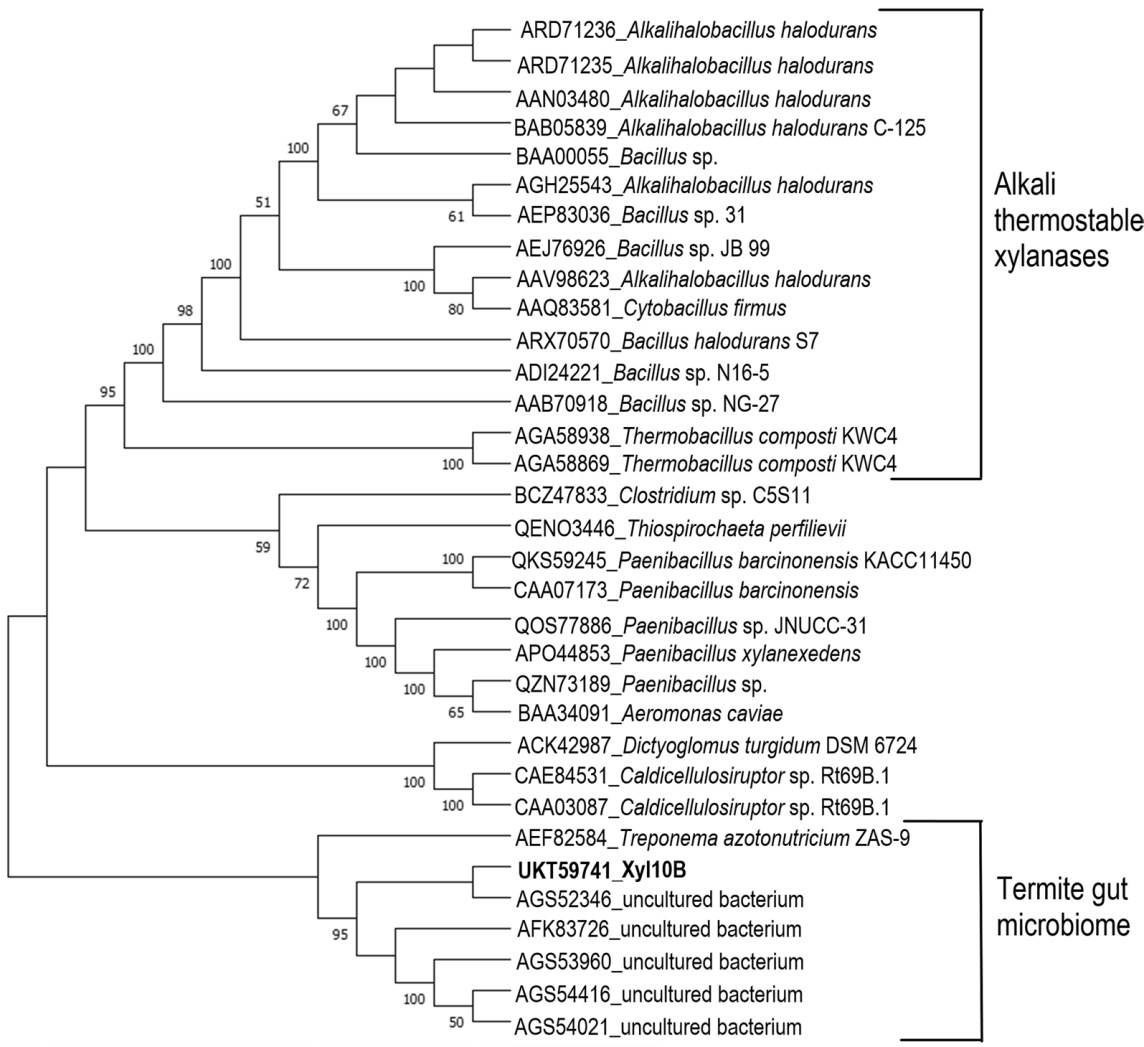
Phylogenetic tree inferred by the maximum likelihood method. The amino acid sequence of Xyl10B is shown in bold

The phylogenetic analysis with the most similar GH10 reference sequences retrieved by BLASTP from the CAZy database revealed that, although Xyl10B grouped with other GH10 bacterial amino acid sequences from termite gut microbiomes, these reference sequences were more distantly related (Fig. 2).

**Fig. 3:**
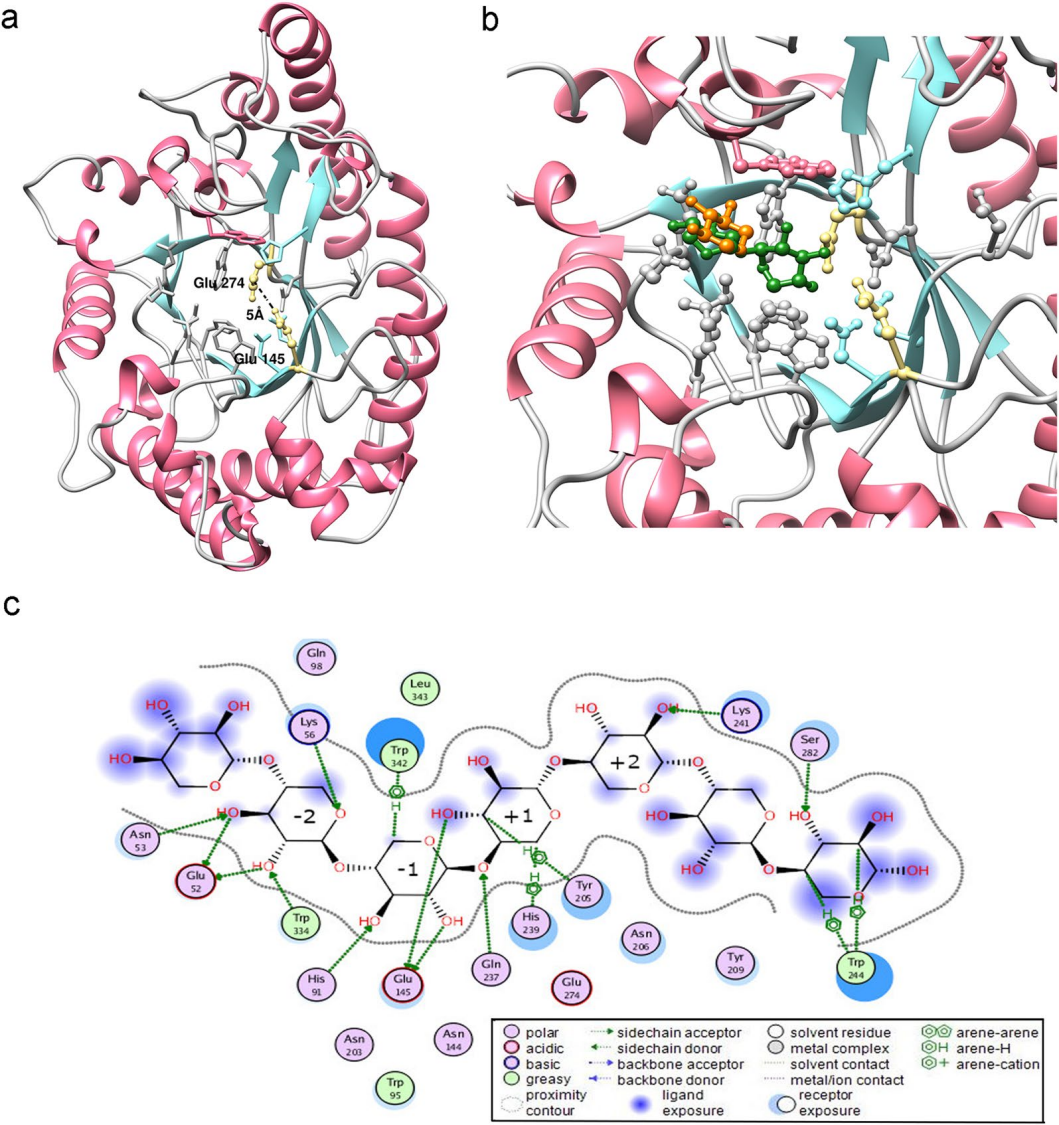
Molecular modeling of Xyl10B. The overall TIM-barrel structure of Xyl10B is shown. The catalytic glutamate residues are indicated (yellow) along with their in-between distance (**a**); zoom showing the top ligand binding residues β-D xylopyranose (orange) and β-D xylose (green) (**b**); schematic representation of ligand interactions (**c**)

As mentioned above Xyl10B molecular models were developed by the I-TASSER and AlphaFold methods, and their quality, after a refining stage (FoldX), was estimated using the structure assessment tool at the SWISS-MODEL Workspace. Specifically, we obtained a MolProbity score of 2 and a QMEANDisCo score of 0.6 for the first AlphaFold model and an improvement for the second FoldX-refined model with a MolProbity score of 1.95 and a QMEANDisCo score of 0.7. The MolProbity score is expressed as a single number that reflects the crystallographic resolution of the model, where a lower score means a better model (Chen et al. 2010). According to references, the above-mentioned quality scores of the AlphaFold raw and FoldX-refined models are enough for the current analysis of Xyl10B functional residues (Baker and Sali 2001). In that sense, the structure of Xyl10B corresponds to the canonical (β/α)8-barrel structural fold (known as TIM barrel), which is common to many glycosyl hydrolases. Regarding the structure and sequence alignment, Xyl10B contains the key residues Glu52, Lys56, His91, Trp95, Asn144, Glu145, Asn203, Asn206, Gln237, His239, Glu274, Trp334, and Trp342, which are conserved in the functional motif of the GH10 family (Tian et al. 2018). The active site is composed of several aromatic residues that line the long active-site groove and are responsible for the ligand-binding function. In the model structure, Glu145 and Glu274 can be identified as the catalytic acid/base and the nucleophile residues, respectively, according to the conserved sequence and their topology. The side chains of the catalytic glutamate residues are at a distance of 5 A˚ (Fig. 3a). Finally, the top predicted ligands for the Xyl10B model were β-D xylopyranose and β-D xylose (Fig. 3b). According to the prediction analyses, the enzyme has the glutamate catalytic residues located at the C terminus of β-sheets 7 and 4, the acid/base Glu145 and the nucleophile catalyst Glu274, respectively. The active site located in a shallow groove, at the C-terminal side of the barrel β-strands, can be divided into two regions (glycon and aglycon), which include several subsites. For Xyl10B, the − 2 and − 1 subsites in the glycon region contain conserved amino acid residues with a predicted key role in substrate recognition. Specifically, the residues Glu52, Lys56, His91, Glu145 and Trp334 established several hydrogen bond interactions with the xylose moieties (Fig. 3c).

Concerning the optimal alkaline activity at high pH, we assessed the number of surface- exposed (> 30%) negatively and positively charged residues, by following an approach detailed by Mamo et al. (2009). Compared to high and non-alkaline active xylanases, Xyl10B has a high percent composition of acidic residues, which results in high ratio of negatively to positively charged residues (Table 1).

**Table 1:** Comparative analysis of the number of surface amino acids composition of Xyl10B with a high and moderate alkaline active GH10 enzymes

		High	Moderate	
	PDB	2UWF	1HIZ	Xyl10B
D		6	7	10
E		24	10	5
Sum Neg		30	17	15
K		7	20	4
R		5	1	3
Sum Pos		12	21	7
Neg/Pos ratio		2.5	0.8	2.1

2UWF and 1HIZ (Mamo et al. 2009)

*Neg* Negatively charged residues, *Pos* Positively charged residues

### Cloning and heterologous expression of Xyl10B

**Fig. 4:**
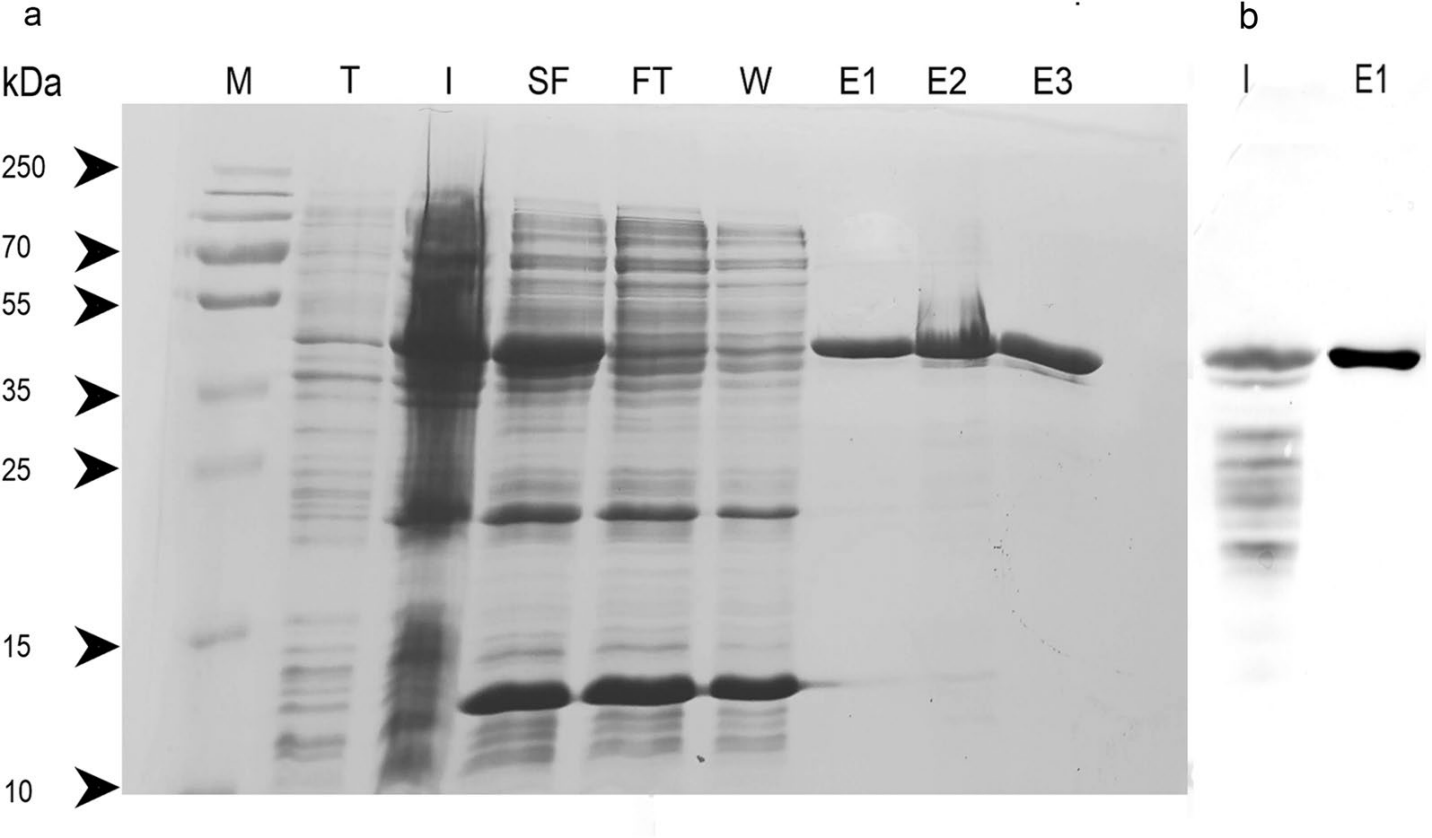
Analysis of purified Xyl10B. Soluble IMAC purification (12% SDS-PAGE) stained with Coomasie blue of Xyl10B. Prestained protein marker (M), T: total protein content of cell lysates without induction, I: total protein content of cell lysates induced with 1 mM IPTG, SF: soluble fraction of cell lysates, FT: flow through, W: wash, E1 to E3: serial elution fractions (**a**), Western blot revealed using anti-His antibody (**b**)

The cloning and recombinant expression of Xyl10B expressed as a 6xHis N-terminal fusion protein without signal peptide yielded a recombinant protein of 46 kDa. This result agrees with the expected size of Xyl10B, as evidenced by the SDS-PAGE and Western blot analyses (Fig. 4a, b).

### Activity characterization of the recombinant Xyl10B

Regarding the hydrolytic activity analyzed on various substrates, Xyl10B showed high activity on beechwood xylan (255 ± 12.33 IU/mg). However, no activity was detected on other cellulosic or hemicellulosic substrates (*p*NPA, *p*NPG, *p*NPX) or CMC (Table 2).

**Table 2:** Substrate specificity of purified Xyl10B

Substrate	Activity	Xyl10B (IU/mg)
Xylan from beechwood	Xylanase	255 ± 12.33
Carboxymethyl cellulose	Endoglucanase	ND
*p*NP-arabinofuranoside	Arabinofuranosidase	ND
*p*NP-glucopyranoside	β-glucosidase	ND
*p*NP-xylopyranoside	Xylosidase	ND

*ND* not detected, *IU* international units (µmol of product/min of reaction)

**Fig. 5:**
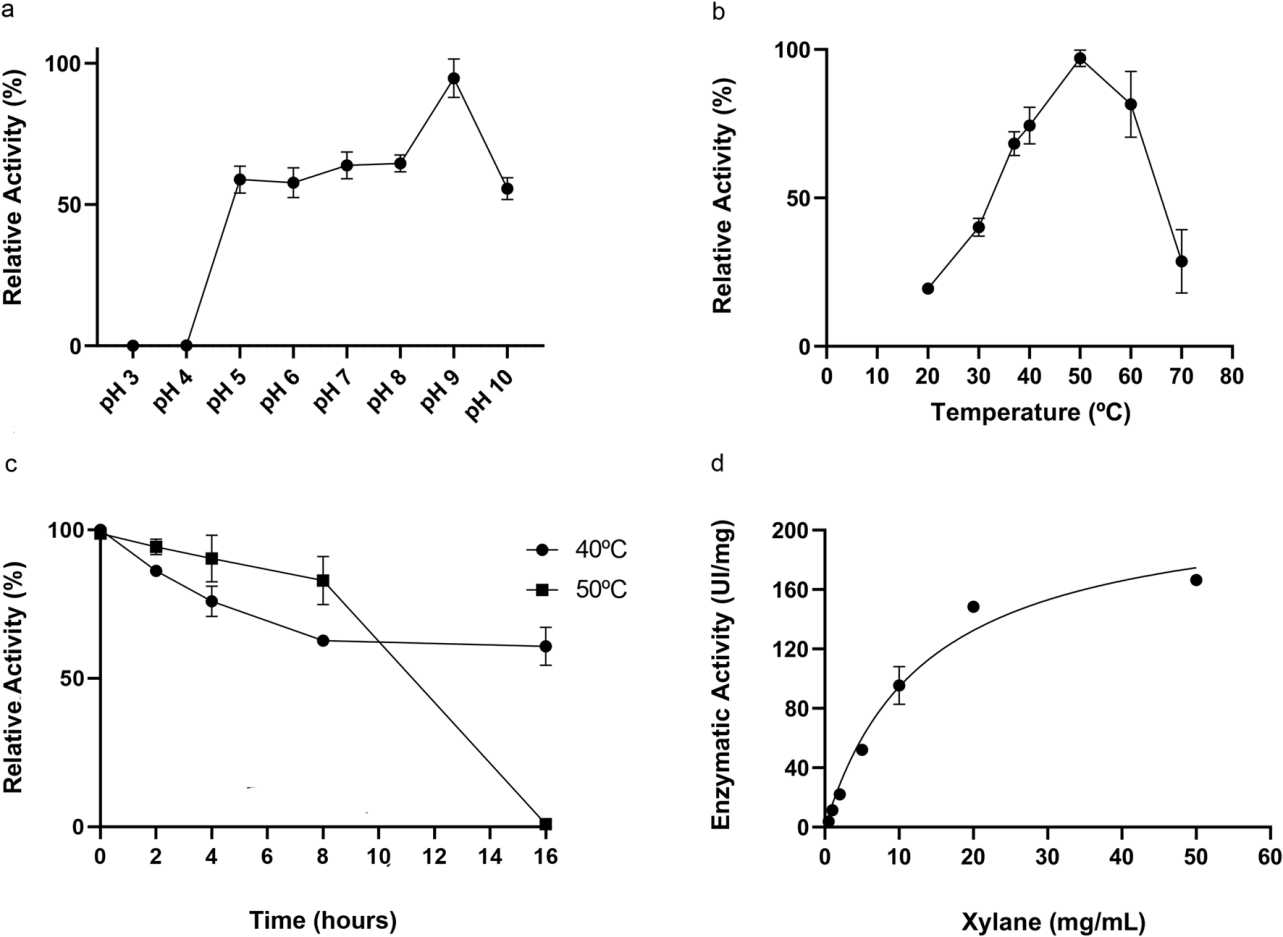
Xylanase profile activity of Xyl10B. Optimal pH value **a**, temperature **b**, thermal stability **c**, and kinetic analysis **d** were evaluated using beechwood xylan as substrate. The results correspond to mean and standard deviations of technical triplicates. Two independent biological replicate assays were made, with equivalent results (IU international units: μmol/min)

According to the endo-1,4-β-xylanase activity analysis, the optimal activity of Xyl10B occurred at pH 9.0 and 50 °C (Fig. 5a, b). Furthermore, Xyl10B showed acceptable activity (> 60%) at broad pH ranges (5–9) and more than 70% between 37 °C and 60 °C. The enzyme maintained more than 80% activity at 50 °C after 8 h and more than 60% activity at 40 °C after 16 h (Fig. 5c).

The Xyl10B kinetic profile on beechwood xylan at optimal pH and temperature values was fitted to a Michaelis–Menten function (Fig. 5d). The Km and Vmax of Xyl10B were 13.49 mM and 221.8 μmol min^−1^ mg^−1^, respectively.

Regarding the effects of some metal ions and chemicals on Xyl10B activity (Table 3), only Cu^2+^ at 10 mM strongly inhibited it, while the other ions evaluated (Ca^2+^, Ni^2+^, Mg^2+^, Mn^2+^ and Zn^2+^) showed no or slight inhibition regardless of the concentration used (concentrations known to inhibit many xylanases). With respect to the other compounds, only SDS strongly inhibited Xyl10B activity, while the others inhibited the activity at a range of 52% to 80% (Table 3). All the substances evaluated significantly inhibit the activity of most xylanases used in industrial applications (Wang et al. 2017a). Finally, Xyl10B was halotolerant, as evidenced by its high activity relative to the control: 93.16% ± 0.12 (NaCl 1 M), 79.55% ± 3.44 (NaCl 3 M) and 64.21% ± 5.46 (NaCl 5 M) (Table 3).

**Table 3:** Effect of metal ions and chemical reagents on the catalytic activity of Xyl10B

Type of additive	Ions or chemical reagent	Relative activity (%)					
		1 mM	10 mM	1 M	3 M	5 M	0.5%
No addition		100	100		100	100	100
Salts	Ca^2+^ (CaCl_2_)	90.99 ± 3.45	96.98 ± 4.02	–	–	–	–
	Cu^2+^ (CuSO_4_)	93.00 ± 5.92	22.89 ± 3.73	–	–	–	–
	Ni^2+^ (NiCl_2_)	94.05 ± 2.22	59.88 ± 3.61	–	–	–	–
	Mg^2+^ (MgCl_2_)	96.59 ± 2.26	105.20 ± 3.05	–	–	–	–
	Mn^2+^ (MnSO_4_)	113.7 ± 10.90	59.88 ± 3.61	–	–	–	–
	Zn^2+^ (ZnSO_4_)	110.84 ± 8.49	107.60 ± 1.41	–	–	–	–
Chelating agent	EDTA	94.64 ± 10.41	82.40 ± 5.77	–	–	–	–
Detergents	SDS	–	–	–	–	–	9.59 ± 2.88
	Tween-40	–	–	–	–	–	74.23 ± 8.08
Organic solvents	DMSO	–	–	–	–	–	80.88 ± 7.57
Reducer and denaturing reagent	β-mercaptoethanol	–	–	–	–	–	52.27 ± 0.58
Salt	NaCl	–	–	93.16± 0.12	79.55 ± 3.44	64.21 ± 5.46	–

### Activity profile of Xyl10B

**Fig. 6:**
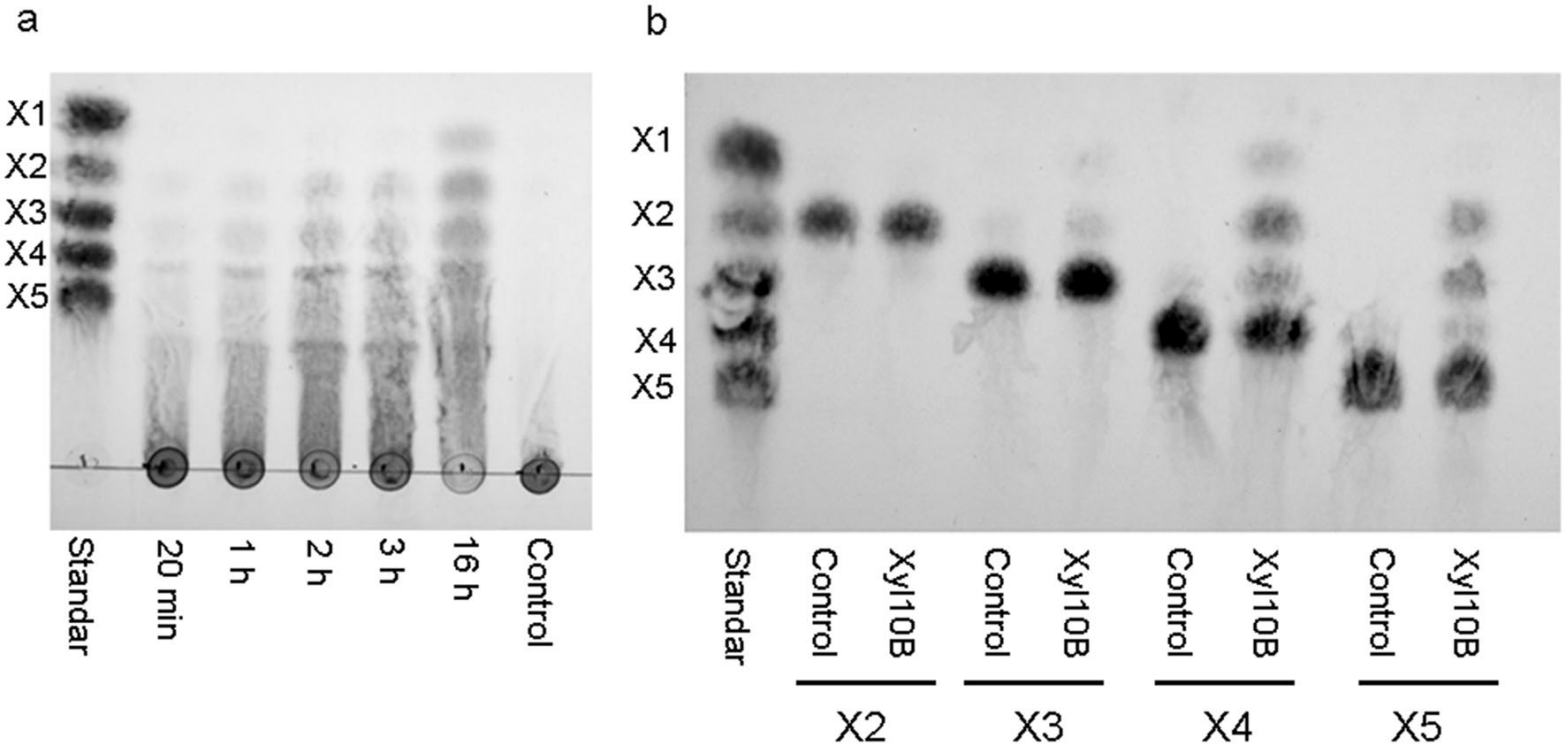
Thin layer chromatography analysis of hydrolysis products of Xyl10B. Time course degradation of 1%beechwood xylan at 20 min, 1 h, 2 h, 3 h and 16 h **a**, hydrolysis pattern of the activity of Xyl10B on short xylooligosaccharides as substrate (10 mM) at 30 min **b**. All assays were performed at 50 ºC pH 9 with 0.65 mg/mL of enzyme. Control: enzyme without substrate, Standard (S) mix of X1: xylose, X2: xylobiose, X3: xylotriose, X4: xylotetraose and X5: xylopentaose (20 mM)

On beechwood xylan, Xyl10B induced an evident increase in the content of xylooligosaccharides over time up to 16 h, by releasing xylose and at least four types of xylooligosaccharides (X2, X3, X4 and X5) (Fig. 6a). With xylooligosaccharides as substrate, in the TLC, Xyl10B was active and hydrolyzed different xylooligosaccharides (X3, X4 and X5). X3 was hydrolyzed to X1 and X2, X4 was hydrolyzed to X1, X2 and X3, and X5 was hydrolyzed to X2 and X3 (Fig. 6b).

## Discussion

In this study, we characterized Xyl10B, a new xylanase, recently identified and selected by a shotgun metagenome sequencing approach by our group (Romero Victorica et al. 2020). Metagenomics is a powerful tool to discover novel bioactive molecules from diverse environments without the limitations of culture (Berini et al. 2017; Fredriksen et al. 2019). This technology has become a revolutionary invention in microbiology because it allows recovering the product of interest through direct cloning from the community DNA and its analysis (Verma and Satyanarayana 2020). However, it should be noted that most researchers have used synthetic genes, and only a few groups have been able to perform successful amplifications from environmental DNA (Ben Guerrero et al. 2020; Joshi et al. 2020; Romero Victorica et al. 2020; Pavarina et al. 2021).

The results of the amino acid sequence analysis here performed showed moderate to low identity with sequences of enzymes of the GH10 family previously identified in the gut microbiota of a wood-feeding higher termite (Liu et al. 2019). Moreover, the phylogenetic analysis revealed that Xyl10B is distantly related to the other GH10 sequences described so far.

From a molecular structure perspective, this protein belongs to GH family 10, with the canonical (β/α)8-barrel fold, which resembles the shape of a salad bowl. The distance of the side chains of the catalytic glutamates (Glu145 and Glu274) suggests that the enzymatic reaction takes place by the retaining mechanism. In addition, the carboxylate group of Glu145 would be held in position by interactions with Gln237 and its acid–base functional role supported by the near aspartates Asp149 and Asp204. Concerning the candidate nucleophile glutamate Glu274, it is surrounded by His91 and His239, an environment that would favor its charged status.

Thus, considering the analysis of its structure and its sequence alignment, Xyl10B has a sequence similar to that of the conserved functional motif of the GH10 family (Tian et al. 2016) and a conserved network of functional residues.

**Fig. 7:**
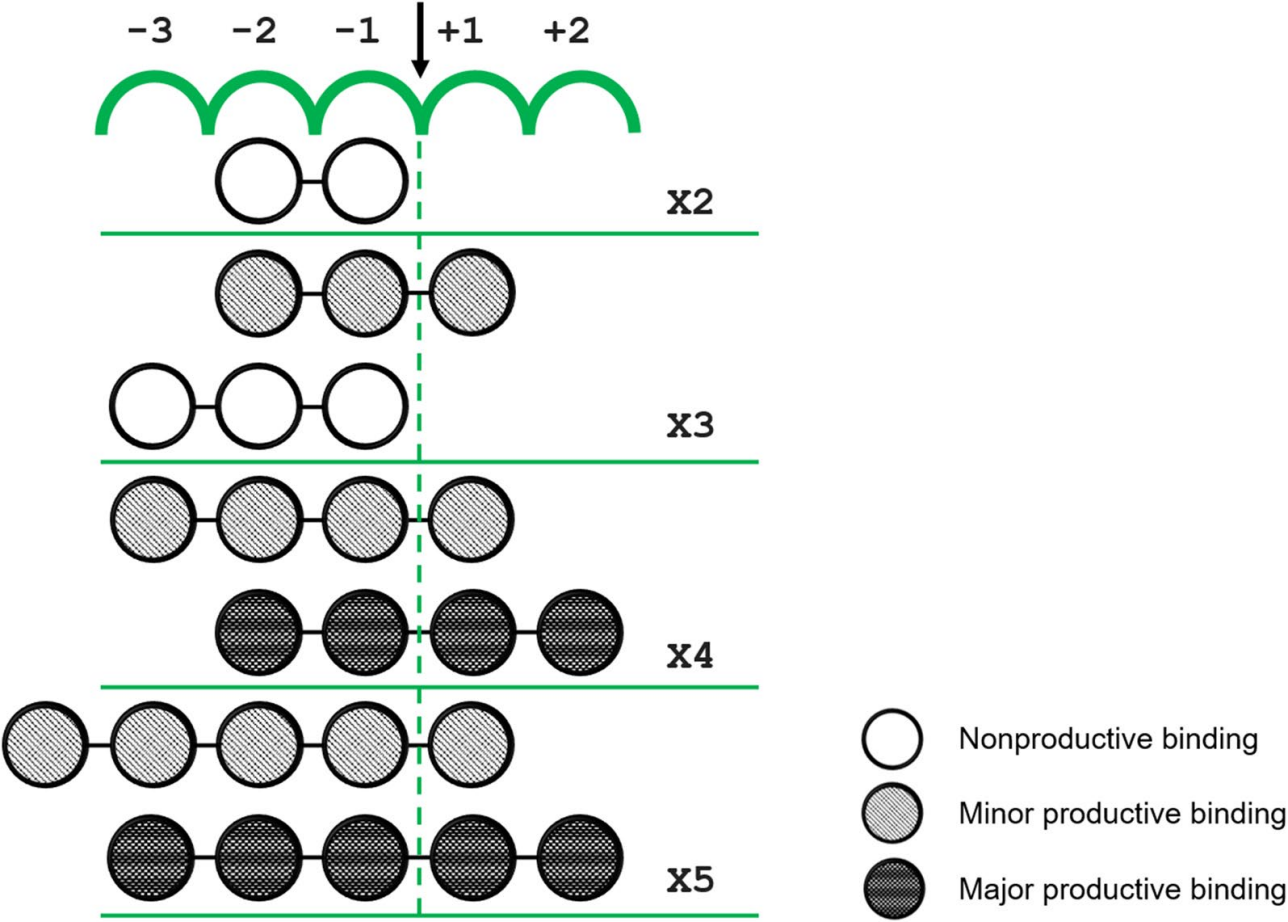
Possible interactions between the subsites of Xyl10B. Bond cleavage occurs between subsites –1 and + 1. Xylobiose: X2, xylotriose: X3, xylotetraose: X4, xylopentaose: X5. Higher grayscale intensity indicates major productive binding

The analysis of the xylooligosaccharides hydrolysis pattern allowed further understanding the mechanism by which Xyl10B recognizes substrates (Fig. 7). Xyl10B was unable to hydrolyze X2, which may be due to the incapacity of X2 to bind at the productive − 1 and + 1 subsites. Concerning X3, Xyl10B exhibited low activity, thus suggesting that only a small number of X3 molecules reach a productive complex between –1 and + 1. In that sense, X3 molecules may be trapped in the region from − 3 and − 1 subsites and form a nonproductive complex, in a manner similar to that observed for X2, where xylose moieties formed stronger interactions with residues at the − 2 and − 1 subsites.

X4 was the first substrate that reflected a more robust enzymatic activity. Cleaved primarily into X2, X4 seems to be the preferred substrate bound between the − 2 to + 2 subsites of Xyl10B. On the other hand, X4 could be bound at the − 3 to + 1 subsites and this would lead to low amounts of X1 and X3.

X5 was hydrolyzed with major productive binding at –3 to + 2, which is a shared feature with X4. This converged in a common core of four subsites (− 2 to + 2), which yielded a more effective function. Other GH10 enzymes have also shown the establishment of strongest interactions at –2 and + 2 moieties (Charnock et al. 1998).

Therefore, previous studies and our protein–ligand interaction analysis, where the four moieties mentioned are the most embraced at the enzyme active site, support the establishment of strongest interactions at -2 and + 2 moieties (Fig. 7).

Except for the xylan specificity, Xyl10B showed no activity towards the other polysaccharides assayed here. Some endo-1,4-β-xylanases have cellulase activity, which can be useful for some industries but detrimental for others. For example, in the paper industry, any cellulose loss is undesirable because it reduces pulp quality (Collins et al. 2005). In the present study, Xyl10B showed no cellulase activity, which makes it a good candidate for the paper industry, e.g. in paper bleaching.

Xylanases are generally inhibited by the end-product, such as xylobiose and xylan hydrolysis products (Mo et al. 2010; Polizeli et al. 2005). In the present study, we found that Xyl10B was not inhibited by xylobiose end-product, because xylooligosaccharides (X3 and X4) can be hydrolyzed into xylobiose and xylose. It should be noted that xylobiose is a product of interest as a prebiotic, since it stimulates the selective growth of intestinal species of *Bifidobacterium* genus, which is important for maintaining a healthy intestinal microbiota (Mo et al. 2010). For this reason, Xyl10B could be useful in the production of xylobiose and would help to reduce the currently very high costs of the industry (Mo et al. 2010).

Xyl10B presented higher specific activity values (255 IU/mg) than other GH10 xylanases identified from metagenomics analyses, such as: Pm25, derived from the gut microbiome of the termite *Pseudacanthotermes militaris* (~ 7.4 IU/mg) (Wu et al. 2021a), denovogenes_5086, from the rumen of Vietnamese native goats (~ 25 IU/mg) (Dao et al. 2021), PW-xyl37, from paper wastewater treatment microbiota (~ 113.7 IU/mg) (Wang et al. 2021), and XynM1, from aquatic habitats of extreme temperature (~ 75.12 IU/mg) (Joshi et al. 2020). On the other hand, Xyl10B presented activity values in the same range as other GH10 xylanases, such as Xyl10E, previously characterized from a termite gut microbiome (~ 288 IU/mg) (Romero Victorica et al. 2020), and Xyn10N18, from bovine rumen (~ 242 IU/mg) (Gong et al. 2013). However, Xyl10B showed lower activity than PW-xy137, derived from paper wastewater treatment microbiota (~ 470 IU/mg) (Wang et al. 2021). As compared with xylanases derived from bacteria, Xyl10B showed higher specific activity than some of them, such as JdXyn10A, derived from *Jonesia denitrificans* (~ 65 IU/mg), CoXyn10A, derived from *Cohnella* sp. (~ 71.16 IU/mg), and XynA2, derived from *Caulobacter crescentus* (~ 1.02 IU/mg) (Jacomini et al. 2020; Hero et al. 2021; Vacilotto et al. 2021). On the other hand, it showed an activity range similar to that of rXylR from *Duganella* sp. (~ 274.7 IU/mg) (Kim et al. 2021) and, in a lower proportion, higher activity than PUL15 from *Prevotella copri* (~ 611.7 IU/mg) (Linares-Pastén et al. 2021) and Xyn30Y5-SLH from *Bacillus* sp. (394.4 IU/mg) (Lai et al. 2021).

The optimal temperature of Xyl10B to degrade beechwood xylan was 50 °C and the optimal pH value was 9. These characteristics make this enzyme an interesting candidate for the pulp and paper industries, since the procedures in these industries require thermo-alkaliphilic enzymes to resist the high temperature and alkaline pH reached (Collins et al. 2005; Dheeran et al. 2012; Verma and Satyanarayana 2020). Besides, given that this enzyme remains active in a pH range of 5–9 (60%), it is applicable to overcome the harsh conditions of the biofuel synthesis processes, such as the increases in pH and salt concentration (Amoozegar et al. 2019).

In this regard, Xyl10B also showed a suitable activity at high salt concentrations. This halotolerance of Xyl10B is an advantage for many industrial applications. In recent years, salt-tolerant xylanases have become interesting candidates because of their potential use in several downstream processes such as bioethanol production, processing of seafood/feed, aquaculture, paper industry and marine wastewater treatment. The high salt concentrations that are produced in the processes of these industries make halotolerant enzymes mandatory (Liu et al. 2013).

Most of the halotolerant and halophilic xylanases so far reported are either from marine bacteria (Guo et al. 2009; Yu et al. 2019) or from saline soils (Prakash et al. 2009; Zhou et al. 2014; Wang et al. 2017b). It is worth noting that, unlike Xyl10B, no other alkaline xylanase from termites has so far been proven to be halotolerant. On the other hand, several metal ions often inhibit the activity of xylanases (Verma et al. 2013). In the present study, however, the candidate enzyme maintained a suitable activity in the presence of most of the metal ions assessed (Ca^2+^, Ni^2+^, Mg^2+^, Mn^2+^, and Zn^2+^). Indeed, this activity remained above 59% in the presence of any of these ions even at high concentrations (10 mM). Thus, these results suggest that Xyl10B is stable and resistant to the main limitations for industrial application.

Most termite gut endoxylanases reported until now are acidic and thermophilic (Brennan et al. 2004; Rashamuse et al. 2017; Liu et al. 2019; Romero Victorica et al. 2020). This could be due to adaptations to the gut environment (Verma 2021). However, several years ago, some researchers reported that the pH in the P1 segment of the hindgut of higher termite guts, especially in the family Termitidae, is strongly alkaline (pH > 9.0) (Bignell and Eggleton 2005). Thus, termite guts could also be useful for the research of alkaliphilic lignocellulose-degrading enzymes (Nimchua et al. 2012).

Therefore, the characteristics of the xylanase Xyl10B, with optimum pH in the alkaline range, the optimum temperature at 50 °C, halotolerant activity, and stability in the presence of several ions and chemical reagents make it a promising candidate to withstand the extreme conditions of several industrial processes.

## Conclusions

The xylanase Xyl10B of the GH10 family, selected from a termite gut microbiome, was heterologously produced. This enzyme showed activity at a broad range of pH values (4–10) and temperatures (37–60 °C), with optimum alkaline pH of 9. The enzyme showed stability and tolerance to several ions and chemical reagents frequently used in the industry. The end products generated after Xyl10B hydrolysis of beechwood xylan were the lower xylooligosaccharides, xylose and xylobiose. In addition, Xyl10B showed unusual enzymatic activity against small xylooligosaccharides, such as xylotriose. All these characteristics make this enzyme valuable for biotechnological processes. Our findings provide basis for further studies for the development of novel enzymes for several industrial applications.

### Supplementary Information

**Additional file 1: Figure S1.** Variation in the control treatments across four independent experiments. The colors of the data points correspond to different experiments. A short degree of noise (jitter) was added to improve the separation between data points. The lower and upper boundaries of the box represent the 75% and 25% percentiles (first and third quartiles) respectively. The coefficient of variation (standard deviation x 100 / mean) was 17.5%.

## Data Availability

All data generated or analyzed during this study are included in this published article and its supplementary information files.

## Abbreviations

Asn: Asparagine
Asp: Aspartic acid
BCIP: 5-Bromo-4-chloro-3-indolyl-phosphate
BLASTP: Basic Local Alignment Search Tool for protein sequences
BSA: Bovine serum albumin
CAZy: Carbohydrate-active enzyme database
CMC: Carboxymethyl cellulose
DMSO: Dimethyl sulfoxide
EDTA: Ethylenediaminetetraacetic acid
GH: Glycoside hydrolase
Gln: Glutamine
Glu: Glutamic acid
His: Histidine
IPTG: Isopropyl-β-D-thiogalactopyranoside
IU: International units
Km: Michaelis–Menten constant
Lys: Lysine
NBT: Nitro blue tetrazolium
NCBI: National Center for Biotechnological Information
Ni–NTA: Nickel-charged nitrilotriacetic acid
PDB: Protein Data Bank
pNP: *p*-Nitrophenol
pNPA: 4-Nitrophenyl α-L-arabinofuranoside
pNPG: 4-Nitrophenyl-β-D-glucopyranoside
pNPX: 4-Nitrophenyl-β-D-xylopyranoside
P1: First proctodeal segment
SDS–PAGE: Sodium dodecyl sulfate–polyacrylamide gel electrophoresis
TLC: Thin layer chromatography
Trp: Tryptophan
Vmax: Maximum velocity achieved by the system at maximum substrate concentration
X1: Xylose
X2: Xylobiose
X3: Xylotriose
X4: Xylotetraose
X5: Xylopentaose
