# Supplementary figures and images for: Characterization of a novel GH10 alkali-thermostable xylanase from a termite microbiome
Source: Bioresour Bioprocess. 2022 Aug 17;9(1):84. doi: 10.1186/s40643-022-00572-w (PMC10992782; doi:10.1186/s40643-022-00572-w)

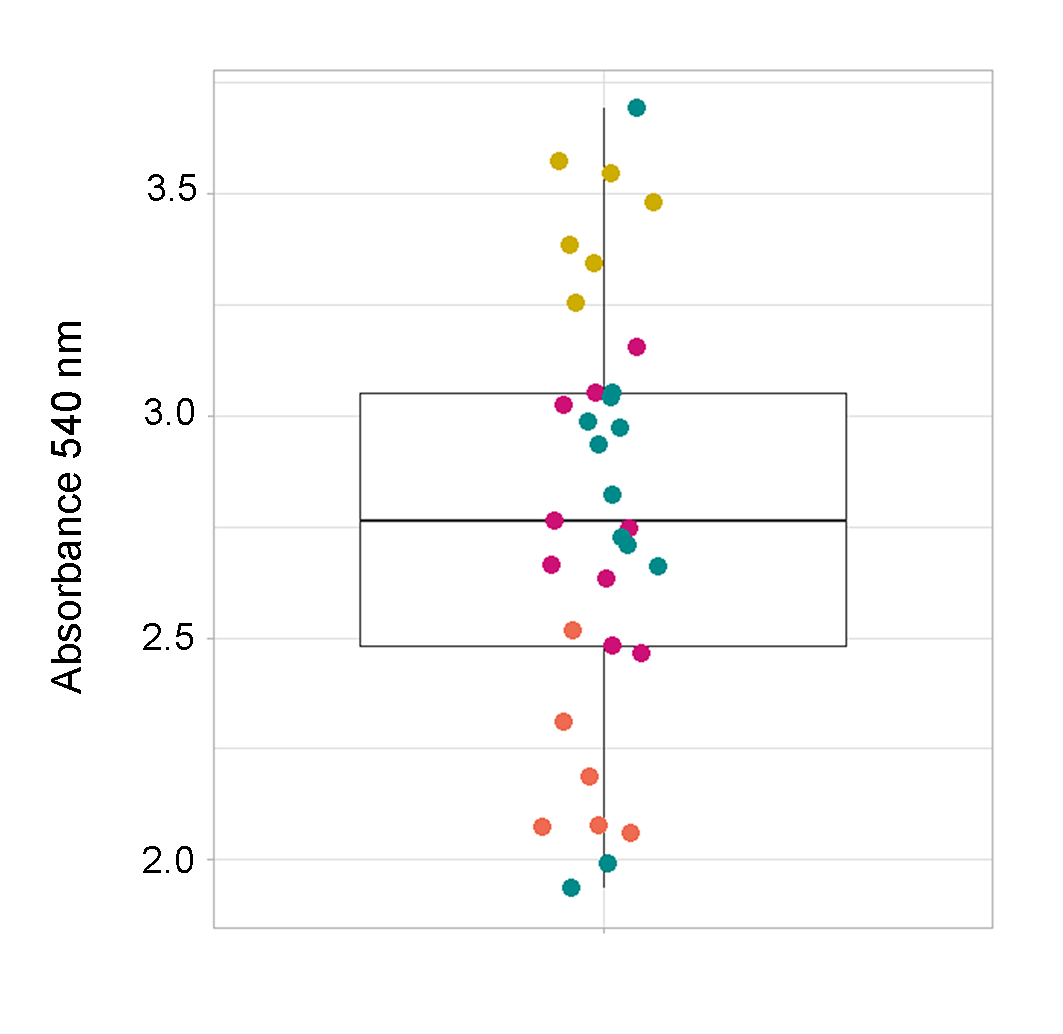

Supplement: Supplementary file 1 — Additional file 1: Figure S1. Variation in the control treatments across four independent experiments. The colors of the data points correspond to different experiments. A short degree of noise (jitter) was added to improve the separation between data points. The lower and upper boundaries of the box represent the 75% and 25% percentiles (first and third quartiles) respectively. The coefficient of variation (standard deviation x 100 / mean) was 17.5%. [file 40643_2022_572_MOESM1_ESM.tif]
